# Therapie der Zystitis mit Nitroxolin – NitroxWin: Prospektive, multizentrische, nicht-interventionelle Studie und mikrobiologische Untersuchungen zur Resistenzsituation

**DOI:** 10.1007/s00120-023-02167-5

**Published:** 2023-08-31

**Authors:** Florian Wagenlehner, Michael Kresken, Esther Wohlfarth, Christina Bahrs, Beatrice Grabein, Walter Ludwig Strohmaier, Kurt G. Naber

**Affiliations:** 1 Klinik und Poliklinik für Urologie, Kinderurologie und Andrologie, Justus-Liebig-Universität, Gießen, Deutschland; 2 Antiinfectives Intelligence GmbH, Köln, Deutschland; 3 Institut für Infektionsmedizin und Krankenhaushygiene, Universitätsklinikum Jena/Friedrich-Schiller-Universität, Jena, Deutschland; 4 Klinische Abteilung für Infektionen und Tropenmedizin, Universitätsklinik für Innere Medizin I, Medizinische Universität Wien, Wien, Österreich; 5 Stabsstelle Klinische Mikrobiologie und Krankenhaushygiene, LMU Klinikum, München, Deutschland; 6 Medical School Regiomed, Coburg, Deutschland; 7 Universität Split, Split, Kroatien; 8 Abteilung für Urologie, Technische Universität München, München, Deutschland; 9 Karl-Bickleder-Str. 44c, 94315 Straubing, Deutschland

**Keywords:** Nitroxolin, Zystitis, Acute Cystitis Symptom Score, ACSS, Harnwegsinfektion, Nitroxoline, Cystitis, Acute Cystitis Symptom Score, ACSS, Urinary tract infection

## Abstract

**Hintergrund:**

Nitroxolin zählt entsprechend der AWMF-S3-Leitlinie zu den Antibiotika der ersten Wahl für die Behandlung der unkomplizierten Zystitis (UZ) bei Frauen. Unter Real-world-Bedingungen sollte die klinische Effektivität von Nitroxolin in einer prospektiven, multizentrischen, nicht-interventionellen Studie (NIS) und die Resistenz von *Escherichia coli* gegenüber Nitroxolin überprüft werden.

**Material und Methoden:**

Patientinnen mit UZ und einer Therapie mit Nitroxolin (empfohlene Dosierung 3 × täglich 250 mg über 5 Tage) wurden von Urologen, Allgemeinmedizinern und hausärztlich tätigen Internisten deutschlandweit von April bis Dezember 2022 rekrutiert und über einen Zeitraum von 21–28 Tagen nachverfolgt. Die Diagnosestellung und der Therapieverlauf wurden mit Hilfe des ACSS-Fragebogens und anhand von Laboruntersuchungen (Leukozyturie etc.) beurteilt. Unabhängig von der NIS wurden im Rahmen einer bundesweiten Resistenz-Surveillance im Zeitraum 2019–20 in 23 Laboratorien *Escherichia-coli*-Urinisolate gesammelt und deren Empfindlichkeit gegenüber Nitroxolin getestet.

**Ergebnisse:**

Von 316 Patientinnen im mittleren (SD) Alter von 57,2 (±20,4; Median 62,5) Jahren, die in die NIS eingeschlossen wurden, war die Therapie zum Zeitpunkt des „test of cure“ bei 193/248 (86,3 %) in der Per-protocol-Gruppe und 193/263 (81,4 %) in der Intention-to-treat-Gruppe klinisch erfolgreich. 96 % der Patientinnen bewerteten die Verträglichkeit von Nitroxolin als „sehr gut“ oder „gut“. Alle 272 getesteten *Escherichia-coli*-Isolate waren Nitroxolin-sensibel.

**Schlussfolgerung:**

Nitroxolin erzielte sehr gute klinische Ergebnisse in der NIS und wies eine sehr günstige Resistenzsituation bei *Escherichia-coli*-Urinisolaten auf. Nitroxolin kann weiterhin als Antibiotikum der ersten Wahl zur Behandlung der UZ der Frau empfohlen werden.

**Graphic abstract:**

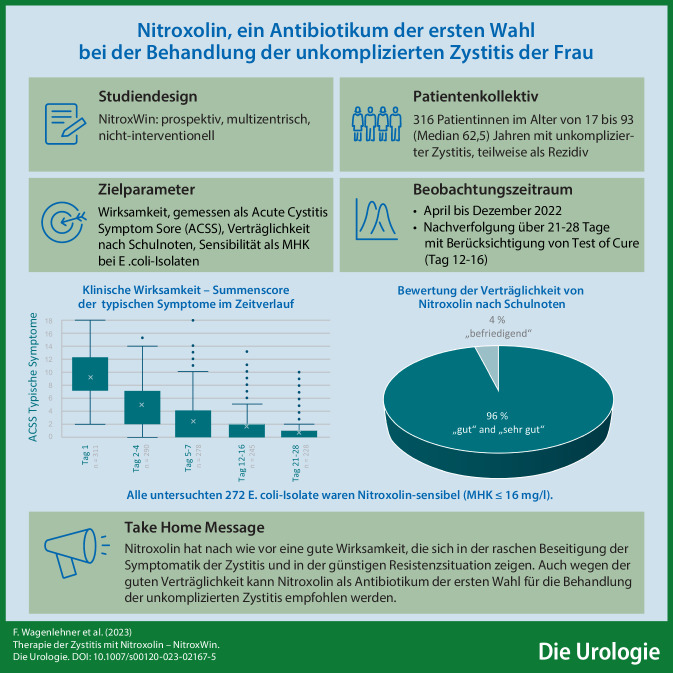

**Zusatzmaterial online:**

Zusätzliche Informationen sind in der Online-Version dieses Artikels (10.1007/s00120-023-02167-5) enthalten. Die Online-Version dieses Artikels enthält weitere Tabellen zur Rezidivhäufigkeit bzw. Dosierung und Therapiedauer von Nitroxolin bei den einzelnen Patientinnen, zu den Keimzahlen und der Mikrobiologie der Urinkulturen, soweit sie bei der Eingangsuntersuchung durchgeführt wurden.

## Abstract (en)

**Background:**

According to German AWMF S3 guideline nitroxoline is recommended as one of the first-choice antibiotics for treatment of acute uncomplicated cystitis (UC) in women. Under real-world conditions the clinical efficacy of nitroxoline should be checked in a noninterventional, prospective and multicenter study (NIS) and the prevalence of nitroxoline resistance in *E. coli* be monitored.

**Materials and methods:**

Female patients with UC treated with nitroxoline (recommended dosage 250 mg tid for 5 days) were included by urologists, general practitioners (GPs), and internists in family medicine throughout Germany from April–December 2022 and followed for 21–28 days. The diagnosis and course of therapy were judged by the Acute Cystitis Symptom Score (ACSS) questionnaire and laboratory investigations (leukocyturia etc). Separately, a nationwide resistance surveillance was performed during 2019–2020 in collaboration with 23 laboratories to collect urinary *E. coli* isolates and test their susceptibility to nitroxoline.

**Results:**

Of the 316 patients with mean (SD) age of 57.2 ($\pm$20.4 [median 62.5]) years who were included in the NIS, 193/248 (86.3%) in the per-protocol group and in 193/263 (81.44%) in the intention-to-treat group were clinically successful. Furthermore, 96% of the patients rated the tolerability of nitroxoline as “very good” or “good”. All 272 *E. coli* isolates tested were susceptible to nitroxoline.

**Conclusions:**

Nitroxoline showed very good clinical results in the NIS, and 100% of the tested *E. coli* urine isolates were susceptible to nitroxoline. Nitroxoline can still be recommended as one of the first-choice antibiotics for treatment of UC in women.

## Hintergrund

Unkomplizierte Harnwegsinfektionen kommen bei Frauen in allen Altersklassen sehr häufig vor [6]. Die AWMF-S3-Leitlinie [8, 14] empfiehlt zur Behandlung einer unkomplizierten Zystitis (UZ) eine antibiotische Erstlinientherapie mit Fosfomycin, Nitroxolin, Nitrofurantoin oder Pivmecillinam (alphabetische Reihenfolge). Trimethoprim hingegen soll empirisch nur noch eingesetzt werden, falls die lokale Resistenzlage für *Escherichia coli* noch bei < 20 % liegt. Fluorchinolone und Cephalosporine sind nicht mehr zur Behandlung einer akuten UZ angezeigt.

## Ziel der Arbeit

Nitroxolin zeigte in vitro v. a. eine sehr gute Aktivität gegen *Escherichia coli* [9]. Das Wirkspektrum erfasst auch andere Uropathogene, einschließlich multiresistenter Stämme [12, 13]. Die aktuelle klinische Effektivität von Nitroxolin bei der Behandlung einer UZ sollte in einer nicht-interventionellen Studie (NIS) geprüft und begleitend dazu die Resistenzsituation bei *Escherichia coli* gegenüber Nitroxolin untersucht werden.

## Studiendesign und Untersuchungsmethoden

Das Design der NIS war offen, prospektiv und multizentrisch. Die Therapieentscheidung zugunsten von Nitroxolin erfolgte, bevor niedergelassene Allgemeinmediziner, hausärztliche Internisten und Urologen Patientinnen mit UZ in die Studie einschlossen. Nicht-schwangere *Patientinnen* (Alter ≥ 18 Jahre) *mit der klinischen Diagnose „akute Zystitis“* und entsprechender Symptomatik wurden in die Studie aufgenommen, sofern bei einer Urinuntersuchung z. B. mit einem Teststreifen eine Leukozyturie (≥ 10 Leukozyten/mm^3^) im Mittelstrahlurin nachgewiesen wurde, entsprechend den Empfehlungen der European Medicines Agency (EMA) und der US-amerikanischen Food and Drug Administration (FDA; [4, 5]).

Die Patientinnen wurden über einen *Zeitraum von 21–28 Tagen* nachverfolgt. Nach der Eingangsuntersuchung am Tag 1 mit umfassender Anamnese, Erfassung der Symptome und der Lebensqualität mittels ACSS-Fragebogen [1, 2], ggf. weiterer Diagnostik (Urinbefund ± mikrobiologische Kultur) und Verordnung von Nitroxolin erfolgten Verlaufskontrollen am Telefon bzw. in der Praxis während der Einnahme von Nitroxolin am Tag 2–4, nach Therapieende am Tag 5–7 bzw. zur Evaluation des anhaltenden Behandlungserfolgs am Tag 12–16 („test of cure“) und am Tag 21–28. Bei allen Kontakten wurde die allgemeine Einschätzung der Patientin bezüglich des Therapieerfolgs (Dynamik), der Schwere der ggf. noch vorhandenen Symptome und der Lebensqualität mit dem ACSS-Fragebogen erfasst. Die Auswahl der Nachbeobachtungstage richtet sich nach den Empfehlungen der EMA und der FDA [4, 5]. Die empfohlene Dosierung zur *Behandlung der UZ mit Nitroxolin* betrug 250 mg 3 × täglich für 5 Tage. Eine davon abweichende Dosierung oder Therapiedauer lag im Ermessen des Arztes und sollte dokumentiert werden.

Wenn beim „test of cure“ (Tag 12–16) die Summe der ersten 5 typischen Symptome (= TS-Score) nicht mehr als 5 betrug, kein Einzelsymptom mit mehr als 1 (= mild) beurteilt wurde und keine Makrohämaturie vorlag, galt die Therapie als erfolgreich (primäres Ziel; [3]). *Sekundäre Ziele* waren die Reduktion der Keimzahl, die Veränderung der Basislaborwerte beim „test of cure“ und die Reduktion des ACSS-Summenscores an Tag 12–16 und Tag 21–28. Für die Auswertung der Leukozyten- bzw. Erythrozytenzahlen wurden die Untersuchungsergebnisse der teilnehmenden Ärzte über die jeweiligen Skalen der verwendeten Testsysteme synchronisiert.

Angestrebt wurde eine *Gesamtzahl* von 200–250 Patientinnen, die deutschlandweit im Zeitraum April bis Dezember 2022 rekrutiert wurden. Jeder an der Studie teilnehmende Arzt konnte maximal 5 Patientinnen dokumentieren. Die ausgewählten Untersuchungen und Verordnungen von Therapien lagen im Ermessen des behandelnden Arztes. Die Datenerfassung erfolgte auf Studiendokumentationsbögen.

Die Patientinnen haben schriftlich ihr Einverständnis zur Teilnahme an der Studie erklärt. Diese NIS wurde in Übereinstimmung mit den *Grundsätzen der Deklaration von Helsinki*, beschlossen auf der 18. Generalversammlung des Weltärztebundes (Helsinki, 1964) sowie entsprechend allen nachfolgenden Änderungen durchgeführt.

*Unerwünschte Ereignisse*, zu verstehen als jedes nachteilige Vorkommnis, das bei einer Patientin unter Nitroxolin-Behandlung auftrat und im Zusammenhang mit der Behandlung stehen könnte, waren innerhalb von 24 h mitzuteilen und zu dokumentieren.

Eine Arbeitsgruppe der Paul-Ehrlich-Gesellschaft für Infektionstherapie hat im Rahmen einer bundesweiten Resistenz-Surveillance im Zeitraum 2019/2020 in Zusammenarbeit mit 23 mikrobiologischen Laboratorien *Escherichia-coli*-Urinisolate von ambulanten Patienten gesammelt und deren Empfindlichkeit gegenüber 10 oral verfügbaren Antibiotika ermittelt [10]. Die Überprüfung der Speziesidentität und die Bestimmung der minimalen Hemmkonzentrationen (MHK) erfolgten in einem Zentrallabor (Antiinfectives Intelligence, Köln). Die MHK wurden mittels Bouillon-Mikrodilution gemäß ISO-Standard bestimmt und entsprechend den Kriterien des European Committee on Antimicrobial Susceptibility Testung (EUCAST, Version 13.0) interpretiert. Bei den Stämmen, deren *Empfindlichkeit zusätzlich gegen Nitroxolin* geprüft wurde, handelte es sich um eine Teilmenge von 272 *Escherichia-coli*-Isolaten. Isolate mit einer MHK von ≤ 16 mg/l werden als Nitroxolin-sensibel und solche mit einer MHK > 16 mg/l als resistent bewertet.

## Ergebnisse

Eine Gesamtzahl von 316 Patientinnen mit einem mittleren (SD) Alter von 57,2 (±20,4) Jahren (Median 62,5 Jahre, vgl. Tab. 1) wurde in die NIS eingeschlossen.

**Table 1:** 

*Alter (Jahre)*	*n (%)*
Jünger als 20	9 (2,9)
21–40	70 (22,2)
41–60	68 (21,5)
61–80	126 (39,9)
Älter als 80	39 (12,3)
Ohne Altersangabe	4 (1,3)
*Menstruation*	4 (1,3)
*Prämenstruelle Beschwerden*	3 (0,9)
*Klimakterisches Syndrom*	20 (6,3)
*Schwangerschaft*	0
*Diabetes mellitus*	35 (11,1)

Urologen waren bei den teilnehmenden Ärzten mit einer Zahl von 73 Ärzten am häufigsten vertreten, gefolgt von Allgemeinmedizinern (*n* = 15) und Internisten mit hausärztlichem Schwerpunkt (*n* = 1). Bei 108 Patientinnen (34,2 %) war die aktuell auftretende Harnwegsinfektion ein Rezidiv. Davon hatten 41 Patientinnen eine sog. rezidivierende Harnwegsinfektion mit ≥ 3 Erkrankungen im letzten Jahr (Suppl. Tab. 1).

Von 296 der 316 Patientinnen liegen Dokumentationen der verordneten Nitroxolin-Dosierung vor (Suppl. Tab. 2). 169 Patientinnen (53,5 %) erhielten die empfohlene Dosierung von 3 × 1 Kapsel à 250 mg täglich über 5 Tage. Bei den anderen Patientinnen wurde die Therapiedauer verlängert (32,3 %) bzw. die Dosierung gesenkt auf 2 Kapseln pro Tag bei verlängerter Therapiedauer (6,3 %). Diese Anpassungen waren unabhängig davon, ob die aktuelle Erkrankung als Rezidiv eingeordnet wurde oder nicht. Die Patientinnen mit aktuellen Rezidiven waren mit unterschiedlichen Antibiotika vorbehandelt: am häufigsten wurden Cotrimoxazol, Fosfomycin und Ciprofloxacin genannt, gefolgt von Pivmecillinam, Bärentraubenblätterextrakt (Uvalysat), Amoxicillin, Nitrofurantoin und Cefuroxim.

Bei 214 Patientinnen wurde ein klinischer Therapieerfolg (Definition s. oben) bei einem „test of cure“ (Tag 12–16) erreicht, was einem Wert von 86,3 % in der Per-protocol (PP)-Gruppe entspricht (Tab. 2). Wenn 15 Patientinnen, die bis zum „test of cure“ die Teilnahme an der Studie wegen mangelnder Wirksamkeit und Umstellung auf ein anderes Antibiotikum bereits abgebrochen hatten, mitberücksichtigt werden (ITT-Gruppe), lag der Therapieerfolg bei 81,4 %. Berücksichtigt man nur die Patientinnen, die die empfohlene Dosierung von 3 × 250 mg Nitroxolin für 5 Tage eingenommen haben, dann zeigten 128 von 145 (88,3 %) in der PP- und 128 von 154 (83,1 %) in der ITT-Gruppe beim „test of cure“ den zuvor definierten klinischen Erfolg.

**Table 2:** 

	„Per protocol“ (PP)		„Intention to treat“ (ITT)	
	Anzahl (Gesamt)	%	Anzahl (Gesamt)	%
Dynamik (ACSS; ≤ 1)	143 (174)	82,2	143 (189)	75,7
Lebensqualität (ACSS; ≤ 1)	193 (248)	77,8	193 (263)	73,4
ACSS; 5 Symptome, dabei TS ≤ 5 und ES ≤ 1 und ohne Makrohämaturie	214 (248)	86,3	214 (263)	81,4
FDA: 4 Symptome, dabei TS ≤ 4 und ES ≤ 1 und ohne Makrohämaturie	215 (248)	86,7	215 (263)	81,7
EMA: 3 Symptome, dabei TS ≤ 3 und ES ≤ 1 und ohne Makrohämaturie	216 (248)	87,1	216 (263)	82,1

*PP* Patientinnen einschließlich Visite 4 („test of cure“), *ITT* PP-Patientinnen plus 15 Patientinnen, die wegen nicht ausreichender Besserung zuvor ausgeschieden sind, *ACSS* Acute Cystitis Symptome Score, *TS* Summenscore typische Symptome, *ES* Summenscore einzelner Symptome

Bei der allgemeinen Einschätzung des Therapieerfolgs (Dynamik ≤ 1 im ACSS), was 174 Patientinnen beantworteten, bzw. der Verbesserung der Lebensqualität (Erfolg ≤ 1) beurteilen die Patientinnen die Wirksamkeit von Nitroxolin als Erfolg mit 82,2 % in der PP-Gruppe (75,7 % ITT) bzw. 77,8 % PP (73,4 % ITT; Tab. 2). Die FDA berücksichtigt nur 4 (Symptome 1–3 und 5 des ACSS) bzw. die EMA nur 3 typische Symptome (Symptome 1–3) einer Zystitis. Passt man die Wirksamkeitsschwelle auf ≤ 4 gemäß FDA bzw. auf ≤ 3 gemäß EMA an, wobei jedes Einzelsymptom als maximal mild bei Abwesenheit einer Makrohämaturie zu beurteilen wäre, ergibt sich eine Wirksamkeit von Nitroxolin mit 86,7 % (PP) bzw. 81,7 % (ITT) nach FDA-Kriterien und 87,1 % (PP) bzw. 82,1 % (ITT) nach EMA-Kriterien (Tab. 2). Bei der Beurteilung der Lebensqualität liegen die Werte etwa 8 % unter denen, die sich aus der Symptomrückbildung ergeben.

**Figure 1:**
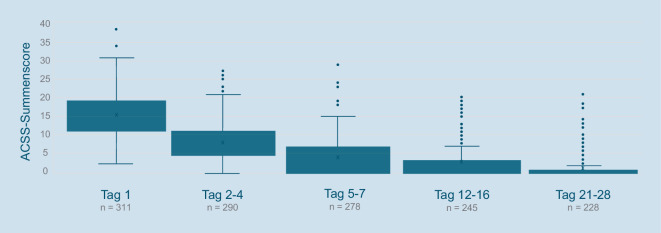

**Figure 2:**
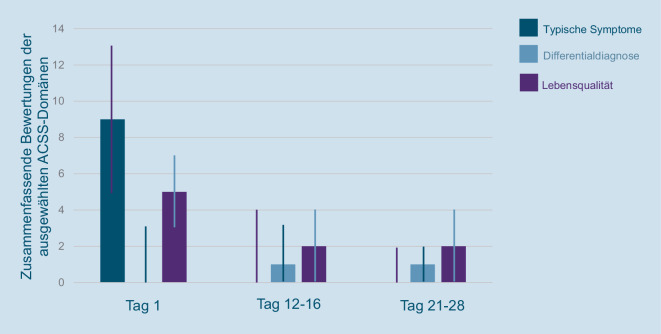

Eine kontinuierliche Reduktion des ACSS-Summenscores konnte über den gesamten Studienverlauf gezeigt werden (Abb. 1). Bei den drei einzelnen Summenscores (typische Symptome [TS], Differentialdiagnostik [DD], Lebensqualität) des ACSS-Summenscores zeigte der TS-Score die deutlichste Reduktion von im Median 9 (± 4) an Tag 1 auf einen Wert von 0 (± 2) an Tag 21–28 (Abb. 2).

Von einer Reihe teilnehmender Ärzte wurden auch labor- und mikrobiologische Untersuchungen durchgeführt. Für die Entwicklungen der Leukozyten- und Erythrozytenzahlen zeigt sich eine tendenzielle Verschiebung von hohen Werten (+++) in Richtung niedrigerer Werte (+ und −, Tab. 3). Diese Entwicklung unterstreicht die Wirksamkeit von Nitroxolin. Bezüglich mikrobiologischer Ergebnisse liegen deutlich weniger Datensätze vor als bei den Patientenbefragungen (Suppl. Tab. 3). Am Tag 1 wurde die Keimzahl im Urin bei 120 Patientinnen bestimmt, wobei 3 Patientinnen eine Keimzahl von 10^2^ KBE/ml und die übrigen Patientinnen eine Keimzahl von 10^4^ KBE/ml und höher aufwiesen. Am zweiten Untersuchungszeitpunkt (Tag 12–16), wenn sich die Infektionssymptomatik bei den Patientinnen, wie aus den anderen Scores ersichtlich ist, deutlich verbesserte und die Mehrzahl nicht mehr unter der Symptomatik der Harnwegsinfektion litt, wurde von den behandelnden Ärzten auf das Anlegen von Urinkulturen in der Regel verzichtet. Bei den vorliegenden Daten kann auch bei den Keimzahlen eine Tendenz Richtung niedrigerer Werte abgelesen werden (Suppl. Tab. 3). Nur bei 38 Patientinnen erfolgte am Tag 1 ein Erregernachweis, wobei *Escherichia coli* am häufigsten angezüchtet worden war (71 %; Supp. Tab. 4).

**Table 3:** 

	Leukozyten (*n* [%])		Erythrozyten (*n* [%])	
Wert	Tag 1	Tag 12–16	Tag 1	Tag 12–16
–	**42 (13,3)**	**175 (70,6)**	*74 (23,4)*	*189 (76,2)*
+	**90 (28,5)**	**39 (15,7)**	*86 (27,2)*	*24 (97)*
++	**56 (17,7)**	**10 (4,0)**	*50 (15,8)*	*12 (4,8)*
+++	**108 (34,2)**	**21 (8,5)**	*61 (19,3)*	*18 (7,3)*
++++	**20 (6,3)**	**3 (1,2)**	*45 (14,2)*	*5 (2,0)*
Total (*n*)	**316**	**248**	*316*	*248*

Einteilung nach Skalen der jeweiligen Combur-Testsysteme: bei Leukozyten (L): + ca. 70 L/µl; ++ ca. 125 L/µl; +++ ca. 500 L/µl; +++ > 500 L/µl – Erythrozyten (E): + 5–10 E/µl; ++ ca. 25 E/µl; +++ da 50 E/µl; ++++ 250 und mehr E/µl

**Figure 3:**
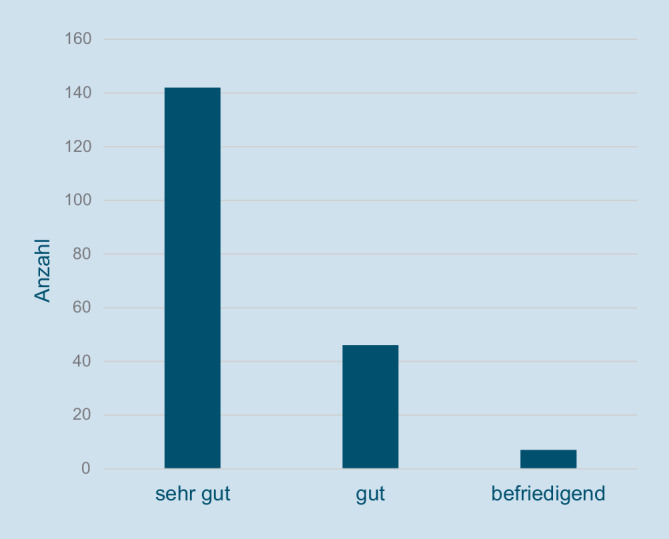

Die Verträglichkeit von Nitroxolin bewerteten 96 % der Patientinnen mit den Noten „sehr gut“ oder „gut“ (Abb. 3). Bei den aufgetretenen Nebenwirkungen (Tab. 4) dominieren gastrointestinale Beschwerden mit 27 Nennungen. 41 Patientinnen gaben im gesamten Studienverlauf an, Nebenwirkungen zu verspüren. Hierbei kam es vor, dass Patientinnen mehrere Nebenwirkungen gleichzeitig nannten. Keine der genannten Nebenwirkungen in der Tab. 4 führte zu einem Studienabbruch. Insgesamt 30 Patientinnen brachen die NIS dennoch vorzeitig ab (Tab. 5). Bei 15 Patientinnen mit mangelnder Wirksamkeit wurde die Therapie auf andere Antibiotika umgestellt. Diese Patientinnen wurden in der ITT-Gruppe als Therapieversager eingestuft. Die Abbruchrate mit 30 von 316 Patientinnen lag bei insgesamt 9,5 %.

**Table 4:** 

Nebenwirkung	Nennungen (*n*)
Magenprobleme, leichte Übelkeit	14
Vermehrter Stuhlgang	2
Diarrhö	7
Bauchschmerzen	3
Verstopfung	1
Kopfschmerzen	5
Müdigkeit	4
Schwindel	3
Urinfärbung	2
Häufigeres Wasserlassen	1
Scheidenpilz	1
Kurzzeitige Gelbfärbung der Haare	1

**Table 5:** 

Abbruchgründe	Tag 2–4	Tag 5–7	Tag 12–16	Tag 21–28
Keine Besserung	5	10	–	–
Diarrhö	1	–	–	–
Juckreiz	1	–	–	–
Herzrasen	–	1	–	–
Schwindel	1	–	–	–
Übelkeit, Erbrechen, Magenbeschwerden	1	2	–	–
Allergie	1	–	–	–
Nachweis Neisseria gonorrhoeae	–	1	–	–
Keine Angabe	–	2	5	1
*Zahl der Abbrüche (Patientinnen)*	*8*	*16*	*5*	*1*

**Figure 4:**
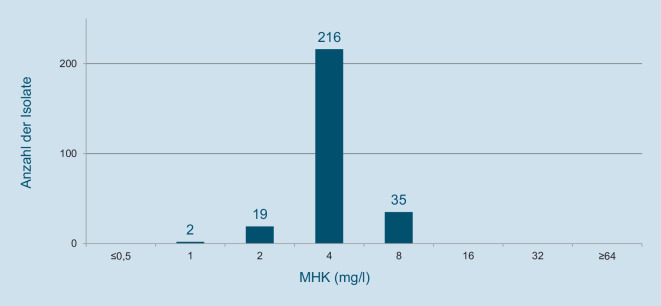

Alle 272 *Escherichia-coli*-Urinisolate aus der bundesweiten Resistenz-Surveillance wurden Nitroxolin-sensibel getestet. Davon zeigten 14 (5,1 %) eine kombinierte Resistenz gegen Amoxicillin, Cefuroxim, Ciprofloxacin und Trimethoprim. Die Verteilung der MHK-Werte von Nitroxolin zeigt Abb. 4. Die MHK50 (50 % der Stämme werden gehemmt) betrug 4 mg/l und die MHK90 (90 % der Stämme werden gehemmt) 8 mg/l.

## Diskussion

Nitroxolin wird in der deutschen AWMF-S3-Leitlinie bei den Antibiotika der ersten Wahl zur Behandlung einer UZ aufgeführt [8, 14], wohingegen die Leitlinie für urologische Infektionen der European Association of Urology Nitroxolin bisher noch nicht berücksichtigt [7]. Die aktuelle AWMF-S3-Leitlinie befindet sich derzeit in Überarbeitung. Bis Ende 2023 wird eine aktualisierte Version zu erwarten sein. Für eine Empfehlung in der Leitlinie ist neben der Resistenzsituation die klinische Effektivität von Bedeutung. Beide Punkte sprechen für Nitroxolin. So fand sich in der mikrobiologischen Studie der Paul-Ehrlich-Gesellschaft für Infektionstherapie von 2019–2020 unter den 272 getesteten *Escherichia-coli*-Isolaten kein einziger resistenter Stamm. Demgegenüber zeigten ca. 5 % eine kombinierte Resistenz gegen Amoxicillin, Cefuroxim, Ciprofloxacin und Trimethoprim [10]. Darüber hinaus bestätigt die vorliegende prospektive Studie die gute klinische Effektivität von Nitroxolin bei UZ mit > 80 % am „test of cure“. In einer Metaanalyse von 4 prospektiv randomisierten Vergleichsstudien, in denen 234 Patientinnen mit Nitroxolin und 232 mit einem der beiden Referenzantibiotika (Cotrimoxazol bzw. Norfloxacin) behandelt wurden, betrug sowohl der mikrobiologische Erfolg (Beseitigung der Bakteriurie) als auch die klinische Effektivität von Nitroxolin > 90 %. Da die klinische Erfolgsrate innerhalb der vorgegebenen Nicht-Unterlegenheitsmarge von 10 % lag, konnte der Unterschied zur Effektivität der Referenzmedikation als klinisch nicht relevant betrachtet werden [11]. Die Verträglichkeit von Nitroxolin und den Referenzsubstanzen war ebenfalls vergleichbar (Nebenwirkungsraten 9,4 % vs. 7,8 %; *p* = 0,36). Die Ergebnisse der durchgeführten NIS bestätigten die gute klinische Effektivität und Verträglichkeit von Nitroxolin.

Die Bewertung der Lebensqualität und der Gesamtbeurteilung durch die Patientin (Dynamik) hängt auch von anderen täglichen Ereignissen als von den bei UZ vorliegenden Symptomen ab. Deshalb wurde nach Analyse auch internationaler Daten empfohlen, als Hauptzielkriterium für die klinische Effektivität die Rückbildung der typischen Symptome heranzuziehen, wobei die anderen Parameter gegebenenfalls zusätzlich beurteilt werden sollten, zumindest wenn Vergleichsuntersuchungen durchgeführt werden [3].

Da es sich um eine NIS handelte, konnten weder bei den Einschlusskriterien bzw. Laboruntersuchungen noch bei der Therapieverordnung weitere Vorgaben gemacht werden. Eine Limitation der vorliegenden Studie war, dass fast ein Drittel der Patientinnen mehr als 5 Tage mit Nitroxolin behandelt wurden. Zur Vermeidung einer möglichen Resistenzentwicklung sollte bei UZ die Therapiedauer maximal 5 Tage betragen. Interessant war die Beobachtung, dass die Therapieerfolgsraten in der Gruppe der Patientinnen, die 3 × täglich 250 mg Nitroxolin für einen Zeitraum von 5 Tagen erhielten, mit 88,3 % (PP) und 83,1 % (ITT) höher waren als die in der Gesamtpopulation.

## Schlussfolgerung

Die Ergebnisse der vorliegenden NIS und der mikrobiologischen Untersuchungen haben gezeigt, dass Nitroxolin bei der Behandlung einer UZ zu Recht in der AWMF-S3-Leitlinie bei den Antibiotika der ersten Wahl aufgeführt wird. Gründe hierfür sind die sehr günstige Resistenzlage von uropathogenen *Escherichia coli* gegenüber Nitroxolin und die guten klinischen Ergebnisse bei der empfohlenen Dosierung 3 × 250 mg täglich für einen Zeitraum von 5 Tagen.

### Supplementary Information




