# Supplementary material for: Therapy of cystitis with nitroxoline—NitroxWin: Prospective, multicenter, non-interventional study and microbiological resistance surveillance
Source: Urologie. 2023 Aug 31;62(11):1186–92. [Article in German] doi: 10.1007/s00120-023-02167-5 (PMC10630225; doi:10.1007/s00120-023-02167-5)
Supplement: Supplementary file 1 [file 120_2023_2167_MOESM1_ESM.pdf]

Suppl. Tabelle 1: Rezidivhäufigkeit bei den 316 eingeschlossenen Patientinnen

**Parameter**	**Anzahl (%)**
Aktuelle HWI mit Rezidiv im letzten Jahr	108 (34,2 %)
- davon ≥ 3 Rezidive im letzten Jahr	41 (13,0 %)
Aktuelle HWI ohne Rezidiv im letzten Jahr	194 (61,4 %)
Keine Angabe	14 (4,4%)

Suppl. Tabelle 2: Im Rahmen der NIS verordnete Nitroxolin-Dosierungen bei 296 Patientinnen, bei denen die Dosierung und Therapiedauer registriert wurde.

**Kapseln/Tag**	**Therapiedauer (Tage)**	**Anzahl der Patientinnen**
2	5	3
2	7	4
2	10	14
2	25	1
3	3	1
3	5	169
3	6	19
3	7	70
3	8	1
3	10	6
3	14	1
3	15	2
3	20	3
4	6	2

Suppl. Tabelle 3: Keimzahlen pro ml im Urin der Patientinnen an Tag 1 und am Tag 12-16

**Keimzahlen/ml**	**Anzahl der Patientinnen**	
**Wert**	**Tag 1**	**Tag 12-16**
0	---	8
10	---	1
100	3	6
1.000	---	2
10.000	13	4
100.000	43	13
1.000.000	59	10
> 1.000.000	2	---
Total (n)	120	44

Suppl. Tabelle 4: Häufigkeit der im Urin nachgewiesenen bakteriellen Erreger bei 38 von 120 Patientinnen mit Urinkultur bei Studienbeginn

Nachgewiesene Erreger	**Primär**	**Sekundär**
Escherichia coli	27	
Enterococcus faecalis	1	4
Klebsiella pneumoniae	1	2
Proteus mirabilis	3	
Klebsiella oxytoca	2	
Staphylococcus saprophyticus	1	1
Streptococcus agalacticae	1	1
Morganella morganii	1	
Enterobacter sp.	1	
Candida glabrata		1
Total (n)	38	9
